# Supplementary material for: MetaRibo-Seq measures translation in microbiomes
Source: Nat Commun. 2020 Jun 29;11:3268. doi: 10.1038/s41467-020-17081-z (PMC7324362; doi:10.1038/s41467-020-17081-z)
Supplement: Supplementary file 10 — Supplementary Data 7 [file 41467_2020_17081_MOESM10_ESM.zip › File2/Confidence_VeryHigh_Taxonomy/84203_out.krona.html]

Javascript must be enabled to view this page.

members
magnitude
magnitudeUnassigned
count
unassigned
taxon
rank

84203\_out

8

2
8
superkingdom

8
phylum
1239

class
8
186801

1
8
186802

SRS1055049\_contig\_number\_contig-100\_1205.1206
order

1
family
541000

genus
1

SRS017521\_contig\_number\_contig-100\_13740.59998
1263

1897042

SRS148159\_contig\_number\_56203
1
species

31979
family
1

1
genus
1485

species
1

SRS013098\_contig\_number\_contig-100\_3269.303012
2305242

family
4
1

SRS019068\_contig\_number\_contig-100\_3850.659221
186803

1
genus
33042

species
1
116085

SRS020233\_contig\_number\_contig-100\_2192.344539

841
1
genus

species
1

SRS018623\_contig\_number\_contig-100\_3128.124505
360807

572511
1
genus

765821

SRS015431\_contig\_number\_63675
species
1
